# Understanding Women’s Pregnancy Intentions, Decision-Making, and Factors Influencing Reproductive Choices After Genital Fistula Repair in Uganda: A Qualitative Study

**DOI:** 10.1371/journal.pgph.0004015

**Published:** 2025-04-11

**Authors:** Mekaleya Tilahun, Hadija Nalubwama, Monica Getahun, Justus K. Barageine, Alison M. El Ayadi

**Affiliations:** 1 School of Medicine, University of California San Francisco, San Francisco, California, United States of America; 2 Department of Obstetrics and Gynaecology, Makerere University College of Health Sciences, Kampala, Uganda; 3 Institute for Global Health Sciences, University of California, San Francisco, California, United States of America; 4 Mulago Specialised Women and Neonatal Hospital, Kampala, Uganda; 5 Department of Obstetrics, Gynecology and Reproductive Sciences, University of California San Francisco, San Francisco, California, United States of America; 6 Department of Epidemiology and Biostatistics, University of California San Francisco, San Francisco, California, United States of America; Federal University, Oye-Ekiti, Nigeria & Wits University, NIGERIA

## Abstract

Female genital fistula is a debilitating injury that may affect as many as two million women globally. While studies have examined women’s fertility intentions in Uganda and sub-Saharan Africa broadly, few have explored the factors influencing pregnancy decision-making among women who have undergone fistula repair. We conducted in-depth interviews with 40 women who had undergone fistula repair. Interviews were audio-recorded, transcribed into English, and coded using a group-developed collaborative coding framework. Oriented by the socio-ecological framework, we reviewed factors contributing to pregnancy desire and decision-making for women who became pregnant and those who did not following fistula repair. Factors influencing pregnancy desire included partner support, financial circumstances, number of children, and health knowledge and perspectives. Women’s own beliefs about their ability to become pregnant and their fears around surgeries and fistula recurrence also influenced pregnancy desire. Participants desiring pregnancy but experiencing infertility expressed various mental health impacts including feelings of hurt, isolation, and yearning, and described infertility stigma. Finally, societal expectations of women to assume childbearing and prioritize home responsibilities influenced participants’ decisions to pursue pregnancy. However, discordance between partners or infertility resulted in various consequences, such as women becoming pregnant to fulfill their partner’s needs, lying to their partner about their pregnancy status, or dissolution of the relationship. A nuanced understanding of pregnancy intentions and decision-making following fistula repair can help inform patient-centered post-repair pregnancy counseling to support the unique needs of women.

## Introduction

Female genital fistula is a debilitating injury that may affect as many as two million women globally, with most cases occurring in sub-Saharan Africa, including Uganda [1,2]. Women with fistula have uncontrollable leakage of urine and/or feces, among other physical symptoms, are heavily stigmatized, and experience high psychiatric morbidity [2]. Most fistulas can be repaired through surgery, but due to the morbidity of this condition and risk of recurrence, optimizing women’s surgical recovery and well-being following surgery is of paramount importance. Providers typically counsel women on several post-operative measures to ensure post-surgical success, including delaying the resumption of sexual intercourse and utilizing contraception to postpone subsequent pregnancy. Yet, post-repair fertility is often a priority for women given the linkages between achievement of fertility intentions and quality of life [3]. Understanding women’s post-repair fertility intentions is a key first step toward supporting women’s agency and ultimately promoting reproductive empowerment [4].

While several studies have examined women’s fertility intentions in Uganda and sub-Saharan Africa broadly [5–11], few have explored the factors influencing pregnancy desire and decisions among women who have undergone fistula repair. Women’s fertility intentions and reproductive experiences following fistula repair vary and are influenced by a multitude of factors at the individual, interpersonal and community levels. For example, one study on the reintegration needs of young women following genitourinary fistula repair in Uganda identified that 85% of women wished to have more children following their repair [7], whereas a Malawian study of fertility outcomes following fistula repair found that two-thirds of women did not desire another pregnancy [8]. Factors contributing to post-repair pregnancy decision-making include women’s knowledge about their fertility status following repair, declining desire or interest in sexual activity, and societal pressure to have children [5,6]. In northern Nigeria, a study identified women’s unwillingness to postpone childbearing following fistula repair, especially in the absence of a living child [3]. Fertility decisions also occur within relationships where women may feel substantial pressure from their partners. These topics were studied in contraceptive research from Uganda which found that male partner support increases contraceptive adoption and that partner influence, often entangled in fears of infertility, is a factor in women’s pregnancy decision-making [12,13].

Our study explored pregnancy intentions, decision-making, and factors influencing reproductive choice among both women who became pregnant and those who did not become pregnant following fistula repair. This qualitative study leverages the socio-ecological framework to draw nuanced insights into pregnancy decision-making following repair across individual, interpersonal, community, and organizational levels. Furthermore, these findings may inform, and guide patient-centered post-surgical counseling focused on the unique needs of women who have undergone fistula repair.

## Methods

### Study design

We conducted a qualitative study to capture the nuanced experiences and perspectives of women’s pregnancy intentions following genital fistula repair. Through in-depth interviews and thematic analysis, we sought to understand the complex interplay of social, cultural, and personal factors influencing reproductive choices. Furthermore, the qualitative design allows for flexibility and responsiveness to emerging themes, ensuring a comprehensive exploration. This qualitative study was embedded within a larger parent explanatory sequential mixed-methods study that sought to understand pregnancy experiences and outcomes following female genital fistula repair, such as factors impacting women’s risk of adverse post-surgical outcomes and experiences following genital fistula repair as well as rates of miscarriage and stillbirth. Study data were collected from December 2019 through January 2023, although the study was paused from March through September 2020 due to the COVID-19 pandemic-related lockdown in Uganda. Qualitative research activities took place from September 2020 – March 2021.

### Study setting and participants

Participants were recruited across Uganda from specialized fistula repair facilities including Kamuli and Kitovu Mission Hospitals, as well as Mubende, Jinja, Hoima, and Mulago Referral Hospitals. During 2010 to 2015, approximately 3000 women had fistula surgery at these facilities [5,14]. At these facilities, fistula repair is provided as an ongoing urogynecological service that is supplemented by several annual 2-week long fistula repair camps to conduct a larger number of fistula repairs. These facilities were selected for [1] provision of fistula-repair surgeries as specialized centers [2], capacity and interest in participating in the study, and [3] availability of eligible participants meeting the study inclusion criteria including history of female genital fistula of obstetric, iatrogenic or traumatic etiology; age 19-49, or above, or emancipated minor; and potential for pregnancy. There were no exclusion criteria. Women were invited for interviews after participating in the quantitative survey, following our purposive sampling strategy below.

### Purposive sampling strategy

We purposively selected 30 participants who became pregnant following their fistula repair and 10 participants who had not become pregnant after their fistula repair. We sampled a total of 40 participants, informed by our prior experiences conducting research and obtaining thematic saturation among this participant population. After conducting these interviews, we were unable to detect new emerging themes, thus concluded that our sample size was adequate. The sampling balanced for post-repair pregnancy outcome [i.e., livebirth, stillbirth, spontaneous or induced abortion], mode of delivery for live and stillbirths [i.e., elective cesarean, emergency cesarean, vaginal], as well as pregnancy status following fistula repair [i.e., pregnancy post-repair, no pregnancy post-repair]. These characteristics were identified from participant screening and quantitative survey data.

### Data collection

Purposively selected potential participants were contacted via phone and invited to participate in the qualitative study. Interviews were conducted in a location where privacy and confidentiality could be ensured [e.g., the participants’ home or a private room at the health facility] and followed COVID-19 protocols for research. We conducted interviews using the preferred language of the respondent by a trained qualitative interviewer familiar with the study population [HN], each lasting1-2 hours. Participants were provided 50,000 Ugandan Shillings [~USD 13.37] to help offset their time and travel costs. In-depth interviews were audio-recorded with participant permission and translated into English for analysis. Semi-structured IDI guides explored pregnancy intentions and decision-making following fistula repair. We asked women about factors influencing their fertility desires and intentions including reproductive decision-making, perceived autonomy, influence from partners and family as well as access to fertility counseling and family planning services. We also asked about the impact of prior pregnancy outcomes on future pregnancy decision-making and on their mental and physical well-being.

### Data analysis

**Fig 1:**
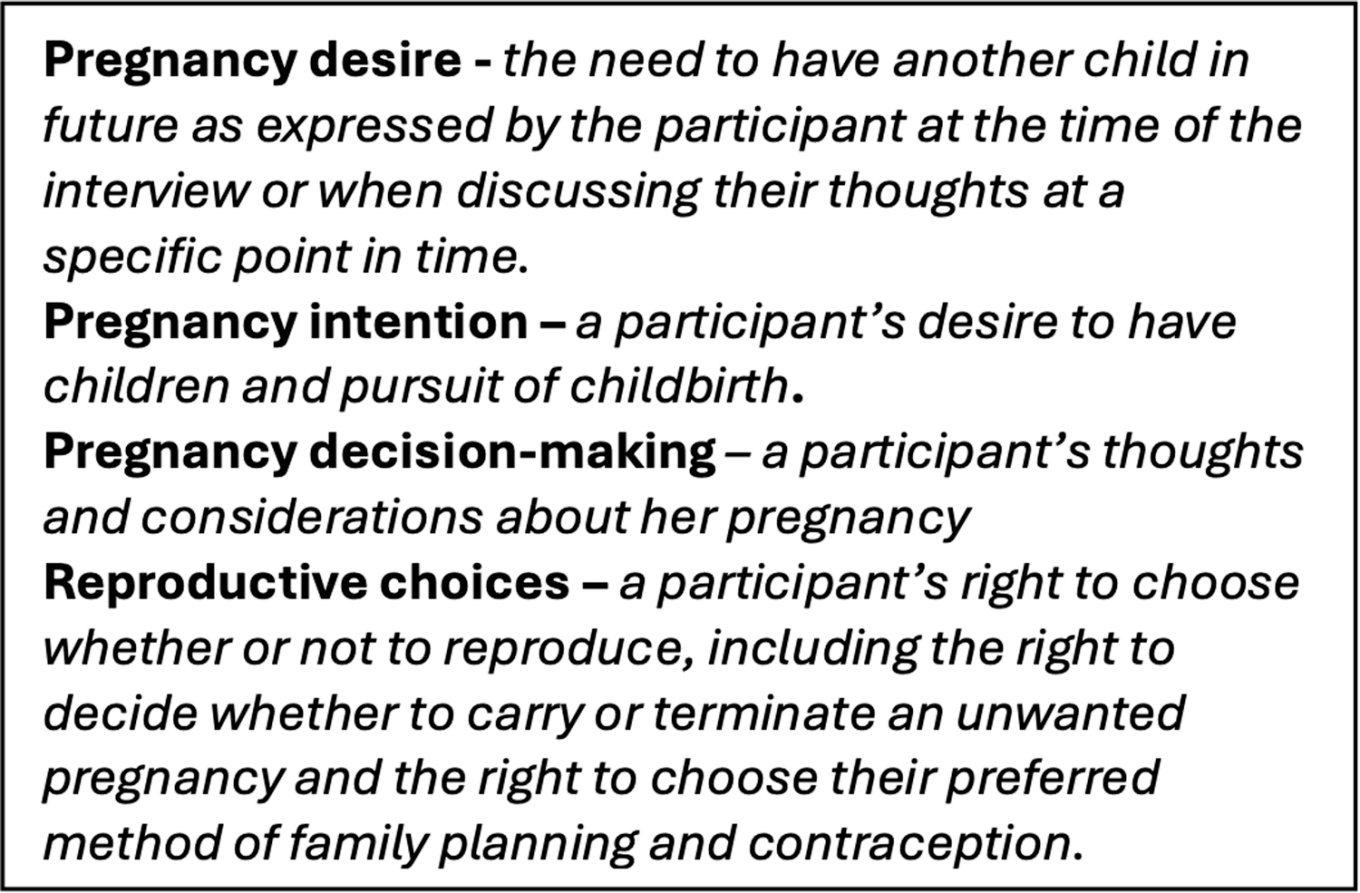
Operational definitions for terms used in results and discussion.

Interviews were translated and transcribed into English for analysis to allow the entire team to be involved. We analyzed data following a two-step process. The first step included coding informed by the interview guide and an initial review of transcripts for potential conceptual categories. Deductive codes, developed based on the IDI guide and prior literature, were applied to the data using Dedoose software. Inductive codes, which emerged organically from the data, were then added to the coding framework and applied to various segments of the text. Six research team members including the Ugandan qualitative researcher [HN], American social epidemiologist [AE], American qualitative researcher [MG], American medical students [MT, SA] and American undergraduate student [MC] collaboratively and iteratively developed the coding framework and coded the transcripts in Dedoose software. The team engaged in iterative reviews of coded segments for concurrence; coding disagreements were resolved by discussion. The lead author [MT] queried codes and developed emerging themes, which were discussed and interpreted iteratively among analysis team members [MT, HN, AE, and MG]. Analysis focused on describing the different dimensions and commonalities of each theme, their distribution across socio-demographic variables, and the patterns and linkages between themes to detect divergent perspectives and experiences by pregnancy desire [i.e., whether the participant desired a pregnancy]. Our themes were then organized utilizing the socio-ecological framework [14] and subsequently refined using aspects of the transtheoretical health behavior change model, which designates iterative stages of change [15]. Further, we have included operational definitions of terms to allow readers to understand their use in discussing findings [Fig 1].

### Ethical approval

Study procedures were reviewed and approved by the University of California San Francisco Institutional Review Board [IRB# 19-27901], the Mulago Hospital Research and Ethics Committee [MHREC# 1674], and Uganda National Council for Science and Technology [HS 2706]. All participants provided written confirmation of informed consent.

## Results

Participants were on average 32 years old, 65% completed primary level education or higher, and 70% became pregnant. Of those who became pregnant, a majority [70%] desired the pregnancy. Of those who did not become pregnant, a majority [70%] did not want to become pregnant [Table 1].

**Table 1:** Sociodemographic characteristics of participants.

Variable	Number	Percentage
**Age** <30 30-40 40^+^	16 16 8	40% 40% 20%
**Marital Status** Married/in partnership Single/never married Widowed Separated	25 2 2 11	62% 5% 5% 28%
**Education Level** None Some primary Completed Primary Some secondary Completed Secondary/higher	2 12 15 10 1	5% 30% 38% 25% 2%
**Employment Status** Self-employed Informal employment Housewife Not employed	7 9 12 12	18% 22% 30% 30%
**Post-repair Pregnancy** Yes No	30 10	75% 25%
**Post-repair Pregnancy Desire** *Became pregnant* Desired pregnancy Did not desire pregnancy *Did not become pregnant* Desired pregnancy Did not desire pregnancy	28 2 7 3	70% 30% 70% 30%

**Fig 2:**
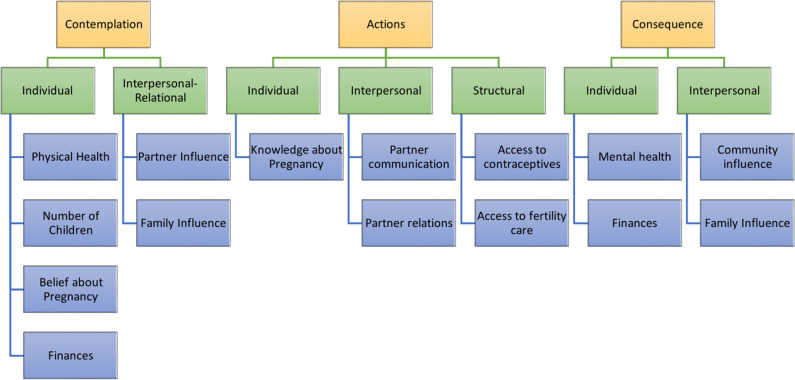
Themes organized using a socio-ecological framework.

We organized themes around factors influencing a participant’s pregnancy intention following fistula repair across individual, interpersonal-relational, community, and structural levels of the socio-ecological framework [Fig 2] [16].

We explore [1] the individual level, including those factors relating to personal beliefs, experiences, and history [2], the interpersonal level, including familial relationships [partners, family members] [3], the community level as the various settings and social relationships with peers and those in the broader community, and [4] structural and health systems-related factors. Results were organized by whether participants desired a pregnancy or not and reflect the nuanced decision-making that surrounds post-repair pregnancies. We describe these within various stages of this decision-making, including contemplation, actions to promote a decision, and consequences of decisions [15], noting that many women often move back-and-forth between these stages, reflective of the many complexities that surround these decisions, and oriented by whether participants desired or did not desire pregnancy.

### Contemplation about pregnancy decision

Individual and interpersonal level factors influenced study participants during the pregnancy contemplation stage. At the individual level, pregnancy decision-making was influenced by personal finances, feelings of having an adequate number of children, physical health, knowledge and beliefs about pregnancy, and perceived societal expectations. At the interpersonal level, pregnancy desire influences included partner support.

### Individual

#### Desired pregnancy.

Individual-level influences on pregnancy desires included women’s beliefs about their physical health and capacity to become pregnant and give birth without issue, views on the adequate number of children, as well as personal beliefs, internalized stigma, and loss of a child.

***Physical Health:*** Some women considered pregnancy as an opportunity to prove their fertility and physical health to others. While the following participant did not want the pregnancy at the time of the interview, she shared that following her repair, she was interested in physiologically proving the possibility of becoming pregnant following fistula:

“*It crossed my mind some time ago when I thought that, maybe I should have one more child as evidence that I got a child after my fistula repair.”* [Participant 1, Age 42, separated, did not become pregnant, pregnancy considered but not currently desired]

Post-repair counseling emphasizes a cesarean delivery for subsequent pregnancies to prevent fistula recurrence. Some participants were encouraged by this counseling and felt assured and motivated to pursue pregnancy. One woman described how she believed a fistula would not recur due to the availability of a cesarean birth:

“I wanted to get pregnant the moment I had healed. When I got to know that I was to have a c-section, I wanted to have children because I wasn’t going to get any more tears.” [Participant 2, Age 30, separated, became pregnant, desired pregnancy]

***Beliefs about the adequate number of children:*** Participants who lost their child at the time of their fistula were emotionally motivated to pursue a post-repair pregnancy to replace their child who had died. These emotional responses were reported among women who lost a child and had no other living children, but not among women who had other children.

*“I wanted to give birth quickly so that I replace my child that I lost […] I needed a child to fill in the gap for my baby that I had lost while giving birth.”* [Participant 4, Age 32, married, became pregnant, desired pregnancy]

***Internalized stigma and infertility:*** Prevailing social pressures and expectations influenced women’s pregnancy intentions. Women reported wanting to become pregnant to avoid what they perceived as a purposeless and meaningless life. These societal expectations were internalized and were especially salient for women with no other children who are experiencing infertility. A woman discussed her challenges with infertility, and recounted her internalized-stigma due to her inability to conceive:

*“I thought that maybe they removed my uterus. But, when I went for a check-up, they told me that it was still there. So, I said that maybe I am barren and wished that I die. Because what is a barren woman doing on earth? I want to have a child because marriage is nurtured by a child.”* [Participant 5, Age 22, married, did not become pregnant, desired pregnancy]

#### Pregnancy not desired.

Among women that did not desire a pregnancy, decision-making was largely attributed to financial insecurity, including supporting their other children and family, as well as concerns about their physical health following fistula repair. Specifically, they had concerns about cesarean birth, fear of fistula recurrence, and perceived fertility.

***Financial insecurity:*** Women shared that the increased costs associated with raising a child, in addition to their existing children and family, deterred them from pursuing a pregnancy following their repair. Others prioritized their livelihoods, noting the difficulty with balancing the responsibilities of raising additional children and their income-generation activities.

***“****I no longer have the appetite to give birth. Whoever tries to tell me about giving birth, I just tell them that I am not giving it a try again. Let me just look for money at the moment, because the moment you try giving birth […] you cannot work.”* [Participant 6, Age 32, married, self-employed, became pregnant, pregnancy not desired]

Women were concerned about the impact another child would have on the care and support that they would be able to provide to their existing children. A participant noted her concern, adding that she was also worried about her daughter potentially becoming pregnant:

*“I wanted to give birth to [another] child but I realized that it wasn’t worth it. My young daughter is in senior six! I realize that I don’t have money to further her education beyond senior six. In fact, I know that men see her wherever she passes, so in no time, she might also get pregnant […] I would relive the situation of taking care of her child, ensuring that he/she gets an education, clothing, food, and everything, and taking care of his/her mother too.”* [Participant 1, Age 42, separated, 3 living children, did not become pregnant, pregnancy not desired]

The sex of existing children also influenced a woman’s pregnancy desire. A participant explained her lack of desire to pursue another pregnancy since she had children of both sexes.

***“****I feel good because my children are my friends. They are not sick, and I have prepared the future for them. So, I look at my family and it is happy. I have a very happy family […] I don’t have any regrets. If I had children of the same sex, I would think about it, I would keep money aside and get pregnant so that I can facilitate my c-section. But now that I have children of different sexes, I quit childbirth. My husband is satisfied because he would have wanted a boy, but I have all of them.”* [Participant 7, Age 36, married, did not become pregnant, pregnancy not desired]

***Fear of cesarean surgery and fistula recurrence:*** Some women did not pursue a pregnancy due to a fear of the cesarean procedure as well as the belief that their fistula would recur.

*“I didn’t want to get pregnant again. Every time he* [partner] *mentioned it, I would tell him, ‘don’t even think about it’...I was so scared* [of C-sections].” [Participant 8, Age 27, married, became pregnant, pregnancy not desired]

Another participant felt fearful that her fistula would recur, which discouraged her from pursuing another pregnancy:

*“After getting that damage* [fistula]*, I got fed up with it, and my husband too got fed up with it. My only reason [for not wanting to get pregnant] was the fistula […] Of course, you get thoughts like, “what if I try childbearing again and get the same problem? Let me just quit” and then God also enables you to do that, and then you suddenly see your husband saying, “let us stop having children.”* [Participant 9, Age 45, married, did not become pregnant, pregnancy not desired]

***Perceived fertility:*** Some women did not believe that they had the physical capacity to become pregnant following their repair, often due to societal misconceptions about fertility. Some reportedly became pregnant despite these beliefs.

*“I heard people saying that to conceive again, one must first resume their menses. That was what I was also waiting to see. [My husband] also didn’t want me to get pregnant but what caused it was following other people’s words, things like; “she cannot get pregnant if she has not resumed her periods.” So, because of that, I failed to start using family planning immediately because I was waiting to first see my period.”* [Participant 3, Age 40, married, some primary education, became pregnant, pregnancy not desired]

### Interpersonal-Relational

Themes influencing pregnancy desire at the interpersonal level included partner fertility preferences and the household support provided by partners.

#### Pregnancy not desired.

***Partner’s fertility preferences:*** A participant’s partner also influenced their pregnancy desire. A woman recounts encouraging her partner to marry someone else to have kids, given her desire not to have children. However, despite these requests, her partner did not remarry, pressuring her instead to pursue additional pregnancy.

*“He [partner] wanted to have a child and he had refused to do what I asked him to do [marry another woman]. If he had done that, I wouldn’t have bothered myself because he would have a new woman, but since he didn’t, I decided to have his children.”* [Participant 8, Age 27, married, became pregnant, pregnancy not desired]

***Lack of partner support:*** For women without resources, the lack of financial support from their partner influenced their pregnancy intentions. A woman below describes how lacking her partner’s support in child-rearing and household responsibilities discouraged her from becoming pregnant, as she believed that he would be unable to care for their future children.

***“****You can never be sure that you are with the right partner. I thought that the moment he made me pregnant, I would have to take care of the pregnancy all alone and yet I didn’t have any money. You see, men of these days, after making you pregnant, one might desert you with your baby.”* [Participant 15, Age 34, separated, did not become pregnant, pregnancy not desired]

### Actions taken to support pregnancy decision

Women faced barriers and facilitators when taking actions to support their pregnancy intentions at the interpersonal and health systems levels. We observed reproductive agency among women who did not desire pregnancy following repair and were supported in their decisions by changing partners. At the health systems level, access to contraceptives supported women’s desire not to pursue pregnancy while those with pregnancy intentions were supported by access to fertility specialists.

### Interpersonal-relational

#### Pregnancy not desired.

***Changing partners:*** Pregnancy desire discordance between women and their partners led to partnership dissolution. A woman shares how she utilized family planning to delay pregnancy, which upset her partner and led to their separation:

*“He wanted me to get pregnant soon...After like a month of us resuming intercourse and I told him no. What I did was that I got on family planning immediately when I left home. He was mad at me and started quarreling and that was the reason as to why we separated... it was me that decided to let go.”* [Participant 10, Age 26, separated, became pregnant, pregnancy not desired]

Another woman describes how she wanted to delay pregnancy to allow her fistula to heal, which reportedly led to her partner leaving, having internalized his friends’ views on his wife’s infertility:

*“[After the fistula repair], he started hanging out with his friends and they started telling him that I won’t be able to give birth. So, he started changing gradually but he knew everything about my fistula.... He agreed to [not having sex] and even found a new wife. Men are not patient, so he found another wife and I endured it because I wanted to heal very well.”* [Participant 11, Age 23, widowed, became pregnant, pregnancy not desired].

### Structural

#### Desired pregnancy.

***Pursuing fertility specialty care:*** Participants experiencing infertility sought fertility specialty care, which was reported to facilitate pregnancy desires. A woman shares how she was considering fertility specialty care to treat her infertility but was ultimately discouraged due to the high costs. She did not share this information with her husband out of consideration for him needing to save money for his other children.

*“I went there [fertility specialist] and they sent me for imaging to see what the problem was […] I failed because it was too costly for me. They told me that I should go with my husband to extract his sperm so that they inject it inside me. However, I didn’t have the money and my husband has other children, so I couldn’t tell him. They also needed millions of money.”* [Participant 13, Age 44, married, did not become pregnant, desired pregnancy]

#### Pregnancy not desired.

***Contraceptive access:*** Participants utilized contraceptives to delay and/or prevent future pregnancy, depending on their desires. A woman shares how she sought a permanent contraceptive option, such as a tubal ligation, to support her desires but found difficulty seeking out this option at health facilities.

*“I wanted them to perform a permanent procedure to stop me from conceiving, but they [doctors] refused. I was so interested. Well, recently, they told me that if I am really interested in it, I would go there, and they give me a referral to go to Mulago hospital because they* cannot perform that procedure.” [Participant 14, Age 33, separated, did not become pregnant, pregnancy not desired]

### Consequence of pregnancy decision

Women reported several consequences of their pregnancy decisions at the individual, interpersonal and community levels. Both participants who intended and did not intend to become pregnant experienced mental health symptoms, i.e., positive effects when they were able to achieve their desires, and negative emotions where they fell short. Women who did not become pregnant reported financial benefits. Family pressure and community norms were reported to influence both groups of women who desired and did not desire pregnancies.

### Individual

#### Desired Pregnancy.

***Mental Health:*** Women who were unable to conceive were negatively impacted, reporting negative effects on their mental health, including their emotions, self-esteem, and self-worth. A woman below describes her hurt at her inability to achieve her pregnancy intentions, including the impact of seeing other pregnant women:

*“It [infertility] makes me restless to the point where you don’t even want to talk about it. You don’t even want anyone to talk about it... It hurts and makes you restless. You think about it and then cry at times. You see a pregnant woman and wish it was me. You imagine how they must be feeling because I was young during my last pregnancy, so I don’t remember how it feels like to be pregnant. I yearn.”* [Participant 13, Age 44, Married, did not become pregnant, desired pregnancy]

Interpersonal differences between partners around fertility desire or capability were a cause of internal and external conflict. Women whose fertility preferences or abilities conflicted with their partner wanting more children experienced significant challenges. Those who could not conceive but desired pregnancy experienced infertility-related stigma, particularly for those women with no children. One participant described the complex dynamics of navigating her infertility with her partner, including how she lied to her partner about her pregnancy status, reflecting self-imposed or societally rooted infertility stigma, power dynamics, fear of abandonment, and financial insecurity:

*“[My husband] wants to have children so much. There are months when I lie to him that I didn’t get my periods, so he takes me to the hospital for a check-up just to find out that I am pregnant. I con him that way. I told him that we are now 3 months pregnant.... I at times lie to him that I need to go to the hospital for antenatal, so he gives me money but I just spend the day at my sister’s and then go back home when it is around 1 pm… I keep on lying because I think that maybe I’ll get pregnant the next month but I don’t.”* [Participant 5, Age 22, Married, did not become pregnant, desired pregnancy]

#### No desired pregnancy.

***Mental health:*** Women experienced positive mental health benefits including relief and appreciation for their existing family at home.

Despite sharing that she had initially desired a pregnancy, a woman shared how she felt relieved at not becoming pregnant due to avoiding the stress and financial insecurity that accompanies pregnancy:

*“[Not pursuing pregnancy] has not affected me. Instead, I think it has protected me... I do not get stressed. Remember that when you are pregnant, you do not work but instead, you are always so stressed thinking, “how am I going to survive?” I thought to myself [...] “what if I was pregnant! I think I would have starved or slept on an empty stomach!””* [Participant 14, Age 33, separated, did not become pregnant, pregnancy not desired currently but desired initially]

***Financial independence:*** Many participants felt that not becoming pregnant allowed their financial independence and provided more time for work.

*“I benefited [from not having children] because if I had another child, my condition would have been very poor. In fact, I wouldn’t have been living in the city anymore... I have searched for money through thick and thin; I have even joined saving groups but my income is too low to save anything. I suffer with paying rent; I search for this child’s school fees and yet his school demands it impatiently. So, I sit there and wonder that if I had added another child, maybe the situation would have been worse.”* [Participant 1, Age 42, separated, did not become pregnant, pregnancy not desired]

### Interpersonal-relational

#### No pregnancy desire.

*Family pressure and lack of support:* Women shared the lack of family support when disclosing their intention to not pursue pregnancy following repair.

*“[My family] is always singing about it! They are always on your case, like, “why don’t you get a man? Why don’t you have children?” They are always telling you that. Your mother and siblings are always like, “you should find a man and have children...of course, it hurts a little because I am also a human being…They always tell you that you should have at least one. That is what they say. Otherwise, no one supports you in not having children.” [Participant 15, Age 34, separated, did not become pregnant, pregnancy not desired]*

Others also shared experiences of concordance and support with their families.

***“****My children say, “Mummy, you should stop. You got that problem last time [fistula], so don’t have any more children.” So, they are also afraid of the other thing [fistula recurrence].”* [Participant 9, Age 45, married, did not become pregnant, pregnancy not desired]

### Community

#### Desired pregnancy.

***Community Opinions:*** Community members were also reported to influence women’s pregnancy intentions, including sharing their views on women’s lack of children. A woman recounts the judgment from community members, including the role that religion plays in attaining her fertility goals:

*“The people in the community also look at you and say, “she doesn’t have a child.” But you have nothing to do about it... Some people say, “it is possible. God can make you get pregnant.” People like my auntie and my siblings. I know God can do it but I just gave up.”* [Participant 13, Age 44, married, did not become pregnant, desired pregnancy]

#### No pregnancy desire.

Community Opinions: While some women did not desire pregnancy immediately, some desired a future pregnancy. Community members expressed negative opinions on these decisions, discouraging women from delaying pregnancies. The woman below shared how she used family planning to prevent pregnancy and ultimately did not feel pressured to have another child just to assuage community opinions:

*“Elder people don’t support certain things and say that for them, they used to give birth to their children without any problem.... Some will keep asking, “When are you having another child?” And I reply, “don’t you see the current economic situation?” Everyone is trying to tell me that it’s time for me to have another child because this one is now grown-up. However, they don’t know what is going on in my life […] People usually say that it’s not right to have only one child with a man.” [Participant 16, Age 34, married, one living child, did not become pregnant, desired pregnancy]*

## Discussion

Across levels of the socio-ecological framework, our findings revealed a complex factors influencing pregnancy desires and decision-making. Some themes spanned across multiple domains and levels, while others were only present in one, and all domains and levels were not always observed to be relevant in each stage of pregnancy decision-making for our study population. Our research supports the need to broadly evaluate influences and competing physical, psychological, social, and economic interests to inform patient-centered strategies to support the reproductive autonomy and care needs of this unique population.

At the individual level, women’s physical health perceptions, including fertility expectations and fears of surgery and fistula recurrence, both positively and negatively influenced post-repair pregnancy interest. Indeed, persistent post-repair fistula symptoms such as urinary leakage, pain or weakness may affect their fertility desires, as has been shown in other settings[6]. Participants in our study expressed comfort in knowing that cesarean surgery could prevent fistula recurrence, whereas others feared surgery or fistula recurrence despite this information, consistent with findings from other research in Uganda and Malawi [6,17]. Women with a prior fistula are at greater risk of fistula recurrence [18], underscoring the importance of access to high-quality pregnancy and childbirth care in post-repair pregnancies. Furthermore, ensuring access to quality care must be combined with enhanced counseling to help women understand the benefits and risks to cesarean surgery. Continued follow-up among this patient population may help us better understand the long-term physical consequences that women experience following fistula repair and their risk factors, further informing counseling and clinical guidelines.

Traditional Ugandan gender norms and expectations consider the husband as the head of the household and decision maker and place high societal values on women to assume childbearing or reproductive roles [19]. Similar to other studies [11,20,21], societal expectations of women to assume childbearing and prioritize household responsibilities influenced pregnancy desires and decision-making. However, desire discordance between partners and/or infertility both had various negative consequences, such as women becoming pregnant to fulfill their partner’s needs, lying to their partner about their pregnancy status, and/or separation. Often, these actions were taken out of fear of abandonment by their partners. Concurrent with previous findings [22,23], separation or abandonment by partners is common after fistula. Participants reported their partners would live with another wife, especially after fistula surgery, leaving fistula survivors without support. Others encouraged their partners to have children with another wife to help facilitate their healing following fistula surgery or chose separation or divorce if there was pregnancy desire discordance, particularly when there were no previous children from the partnership. Because many women with fistula have lower socioeconomic status, they may be more reliant on their partners for social and financial support to meet their various needs [20,21]. While women who separate from their partners may lose both social support and economic capital, their agency in the decision-making process indicates a clear shift away from traditional gender expectations. Establishing and enhancing social support systems after fistula repair may help empower women to achieve independence after fistula repair.

Stillbirths in fistula-causing births occur frequently and women who lost a child during that experience may desire a child to replace that loss [24,25]. The important impact of prior stillbirth on pregnancy desire and timing of next pregnancy observed in our study was also noted in a Nigerian study which found women with no living children were unwilling to delay` childbearing after fistula [3]. In our study, pregnancy timing was also impacted by low fertility knowledge on fertility and the social pressure of infertility stigma. The prevalent stigma surrounding the loss of a child along or social perception that women with fistula are infertile combined with a high value on childbearing due to societal expectations have not only been found important in our study, but in other research on fistula [24,26].

Not all women win our study who desired pregnancy following fistula repair were able to achieve it, with some reporting challenges becoming pregnant. Some women with fistula experience secondary infertility or recurrent spontaneous abortion before or after surgical repair, and while the mechanisms are not completely known, these outcomes are attributed to severe cervical destruction that may occur during prolonged obstructed labor [27]. Desiring pregnancy but experiencing infertility was also associated with mental health morbidity and exacerbated by the general inaccessibility of infertility care in this context. Some participants who experienced secondary infertility sought out IVF and other fertility treatments from specialists; however, none followed through with the care due to the high costs of treatment. This impact and experience of women with fistula in our study is similar to infertility experiences among Ugandan women without fistula, who report distress and grief from unfulfilled desires as well as the inability to afford IVF treatments due to their high costs [28]. Empathetic counseling on the variable fertility experiences women may have following fistula repair, screening for mental health symptoms, and connection to appropriate care can improve women’s experiences. Furthermore, increased accessibility of pre-conception and fertility healthcare resources during all stages of a woman’s pregnancy decision-making process is needed.

In our study, participants did not pursue pregnancy due to a lack of access to economic resources, including money and job opportunities, as not pursuing a pregnancy enabled them to pursue economic opportunities. Women with fistula experience great economic insecurity and often are economically vulnerable even before developing fistula. Economic support and income-generation skills development is recognized as important components of the post-repair rehabilitation process for helping women overcome the economic consequences of fistula and position them for broader improvements in socioeconomic status [29,30]. This is consistent with the priorities of women in our study. A systematic review examined existing rehabilitation and reintegration programs provided adjunct to genital fistula surgery based largely in sub-Saharan Africa and identified various combinations of post-surgical and social reintegration efforts addressing needs demonstrated in the literature including health education, physical therapy, social support, psychosocial counseling, and economic empowerment [29,31,32]. Positive outcomes result from several reintegration approaches, however, studies on the feasibility and outcomes of integration remain a major gap in the literature.

Many participant interviews revealed that pregnancy decision-making does not follow a consistent trajectory and the desire for pregnancy or not to become pregnant was not concrete. Several factors influenced a participant’s preferences and plans that often changed over time since a participant’s fistula and fistula repair. This is a characteristic of pregnancy intention overall and is an important consideration when providing care to this population, as the decision to pursue or not pursue pregnancy is fluid and should be an ongoing discussion with providers throughout the continuum of care [33].

### Strengths and limitations.

We note several limitations in the study. First, our small sample size and originating geography may limit the applicability of our findings. Second, we did not verify current pregnancy or fertility status at the time of the interview, which may have been several years after a participant’s fistula repair. Third, participant responses may have been influenced by social desirability bias. We implemented several strategies to mitigate these limitations. First, patients were interviewed by study staff who had previous contact with patients and built rapport to encourage open feedback. Second, our analysis and interpretation involved a diverse binational study team, including those who collected data, which strengthened the rigor of the study and the validity of our data. Finally, our use of established theoretical frameworks to contextualize our data reinforces the importance of a multi-level approach to understanding and facilitating pregnancy decision-making and desires.

## Conclusion

Our study revealed that factors in individual, interpersonal-relational, community, and structural domains influenced women’s decisions to pursue pregnancy after fistula-repair. Barriers and facilitators include physical health perceptions, fears of surgery and fistula recurrence, traditional Ugandan gender norms and access to economic resources. Continued follow-up and counseling that addresses the complex interplay of physical, psychological, social, and economic factors impacting this population is needed. These efforts should span across a longitudinal time course given the fluid and ever-changing nature of pregnancy decision-making in this population. Furthermore, future studies should examine the long-term consequences of these pregnancy decisions on this population to inform more contextually relevant counseling following fistula repair.
